# A comparison of the sampling effectiveness of acoustic recorder, camera trap and point count methods in sampling nocturnal birds in Afrotropical landscapes

**DOI:** 10.1002/ece3.11389

**Published:** 2024-05-20

**Authors:** Yitmwa Hope Joel, Iniunam Aniefiok Iniunam, Danjuma Filibus Dami, Ulf Ottosson, Adams Adamanyiwa Chaskda

**Affiliations:** ^1^ Department of Zoology, A.P. Leventis Ornithological Research Institute University of Jos Jos‐East Plateau State Nigeria

**Keywords:** acoustic bird survey, camera traps, nocturnal birds, point count, species richness

## Abstract

Conservation decisions for bird diversity in the Afrotropics are often based on ecological studies utilizing diurnal bird species likely owing to difficulties associated with sampling nocturnal birds. It is therefore important to compare the sampling effectiveness of some of the available techniques that can be used in nocturnal bird surveys to guide future long‐term survey efforts. Thus, we compared the sampling effectiveness of point count, acoustic recorder and camera trap for estimating nocturnal bird species richness and also across habitat types. We surveyed 20 points that were spaced at least 500 m apart in November and December 2021 in the Amurum Forest Reserve and its surroundings in Jos‐Nigeria. At each point, we used two camera traps, one at the ground and the other at 2.0 m. We also used one acoustic recorder as well as a 15‐min point count during each survey at each point. We encountered 11 nocturnal bird species, primarily nightjars but also owls. While we did not encounter any species with the camera traps, all 11 species were recorded using the acoustic recorder. All species except for *Ketupa lacteaus* were recorded in point count. Eight species were recorded in the gallery, seven in rocky and nine in savannah. Species richness and estimation using the acoustic recorder and point count were similar across habitat types. We conclude that either point count or acoustic recorders are useful for nocturnal bird surveys in Afrotropical environments. However, the choice of methods should be based on the research questions as some questions may be better answered by a specific method or even a combination of both.

## Graphical abstract

The research compares the sampling effectiveness of three survey methods (camera traps, acoustic recorder and point count) in sampling nocturnal birds (a poorly studied avian group) in Afrotropical landscapes, Nigeria. The result showed that acoustic recorders are as effective as the traditional method (point count) in estimating nocturnal bird richness.

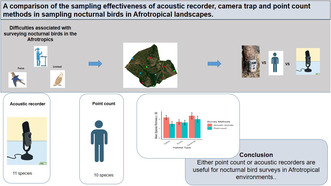

## 1 INTRODUCTION

Most avian monitoring programs and research in the tropics are restricted to diurnal birds (Goyette et al., 2011; Pérez‐Granados & Schuchmann, 2020; Puan et al., 2015). Some of the reasons are that they are easy to detect and are active during the day (Bhattacharje, 2008; Gaunt & McCallum, 2004). The inclusion of nocturnal birds is mostly through incidental observations (Goyette et al., 2011) or restricted to pre‐dusk and dawn censuses (Goyette et al., 2011; Puan et al., 2015). As a result, nocturnal birds in the tropics are amongst the most poorly studied avian groups (Fröhlich & Ciach, 2019) despite all the roles they play in ecosystem functions (Kettel et al., 2018).

Nocturnal birds serve as apex predators that regulate the population of other species such as rodents and insects (Moreno‐Mateos et al., 2011; Rashid et al., 2021) thus serving as agents of biological control (Ghimire, 2016; Zuraidy et al., 2015). Also, nocturnal birds such as owls indirectly reduce the transmission of some deadly diseases and pandemics to humans (Min et al., 2020; Rashid et al., 2021). Furthermore, they are keystone species whose decline in population or extinction can lead to top‐down cascade ecological consequences (Sekercioglu, 2010). Most nocturnal bird species are referred to as ‘management indicator species’ due to their high‐quality habitat requirements, such that conserving them means conserving other species, thereby fitting in as umbrella species (Mikkola, 2017; Simberloff, 1998). In addition, due to the likelihood of dietary niche overlap, changes in the number and variety of the nocturnal bird population may indirectly affect the size and diversity of the diurnal bird population (Jayson & Sivaram, 2009). Considering the roles played by these species, it is vital that effective methods are employed to monitor the species. There have been several attempts to establish a standardized and reliable method for sampling these nocturnal species. These methods have ranged from traditional distance sampling methods such as point count (Bibby et al., 2000; Leach et al., 2016) to several other technologically advanced methods such as radio telemetry and GPS tracking (Evens et al., 2017). These methods have paved the way for biodiversity monitoring in different ecosystems and have encouraged the study of nocturnal wildlife (Aide et al., 2013; Sugai et al., 2019; Yack et al., 2013).

Amongst the survey methods are the broadcasting method, i.e. the use of playbacks (Kemp et al., 2009), the distance census techniques (Bartolomei et al., 2013; Dayananda et al., 2016; Moreno‐Mateos et al., 2011), the use of automated acoustic recorders (Docker et al., 2020; Goyette et al., 2011; Raymond et al., 2020) and other non‐invasive sampling methods such as the use of molecular methods like DNA analysis from faecal and feather samples (Pedroso et al., 2018). Others involve the use of geolocators for species‐specific studies (Evens et al., 2017). However, the vast majority of these studies were conducted in the temperate zone with none on nocturnal birds in the Afrotropics. Also, none of these studies evaluated these methods simultaneously with a view to assessing their effectiveness in surveying nocturnal birds.

Night‐time surveys present challenges to observers' safety in addition to the problem of low visibility (Pérez‐Granados & Schuchmann, 2020; Puan et al., 2015). Most nocturnal species are secretive, crepuscular and cryptic and are active at a time when access to sites is quite challenging often due to security issues. This has resulted in the implementation of several conservation management objectives such as the management of habitats and protected areas based on diurnal bird monitoring only. However, details on the validity of these approaches for use in determining nocturnal birds' abundance and richness are what conservation planners most require to enhance the quality of data for decision‐making and help in the development of proper and effective monitoring programs.

Nigeria is lacking a comprehensive inventory for nocturnal birds; however, evidence suggests a rich assemblage of owls, nightjars and other species is found across the country (Elgood et al., 1994; Okosodo et al., 2016). Some of the recorded species are African scops owl (*Otus senegalensis*), Standard‐winged nightjar (*Caprimulgus longipennis*), African wood owl (*Stix* Woodfordii) and Western barn owl (*Tyto alba*) amongst others (Borrow & Demey, 2014; Elgood et al., 1994). Owing to the difficulty in sampling these species, their ecology remains poorly understood.

Our primary goal was to provide information on effective survey techniques for nocturnal birds in an Afrotropical environment. We particularly sought to (i) compare bird richness using three survey methods – acoustic recorder, point count and camera trap, (ii) compare the sampling effectiveness of the survey methods in various habitat types in a Guinean savannah (Savannah woodland) ecological zone which is the most extensive vegetation in the middle belt Nigeria (Adedibu et al., 2022) – we define sampling effectiveness of a method as the potential for a method to capture or record all species expected of it and detection as the sum of the number of observations of all species by each method (iii) and provide recommendations for nocturnal bird survey in the Afrotropics.

We expect that acoustic recorders and camera trap survey methods will be more effective in sampling nocturnal birds as they are non‐intrusive compared to the traditional point count method (Buxton et al., 2018; Fontúrbel et al., 2020). Also, we expect the sampling effectiveness of the different methods to vary between habitat types owing to differences in habitat characteristics or structure as such structures may compromise the detection of some species (Fontúrbel et al., 2020). For instance, dense vegetation might alter observation resulting in low visibility during point count surveys. It can also obstruct the camera field of view and interfere with the transmission of sounds resulting in difficulty in species identification.

## 2 MATERIALS AND METHODS

### 2.1 Study area

**FIGURE 1:**
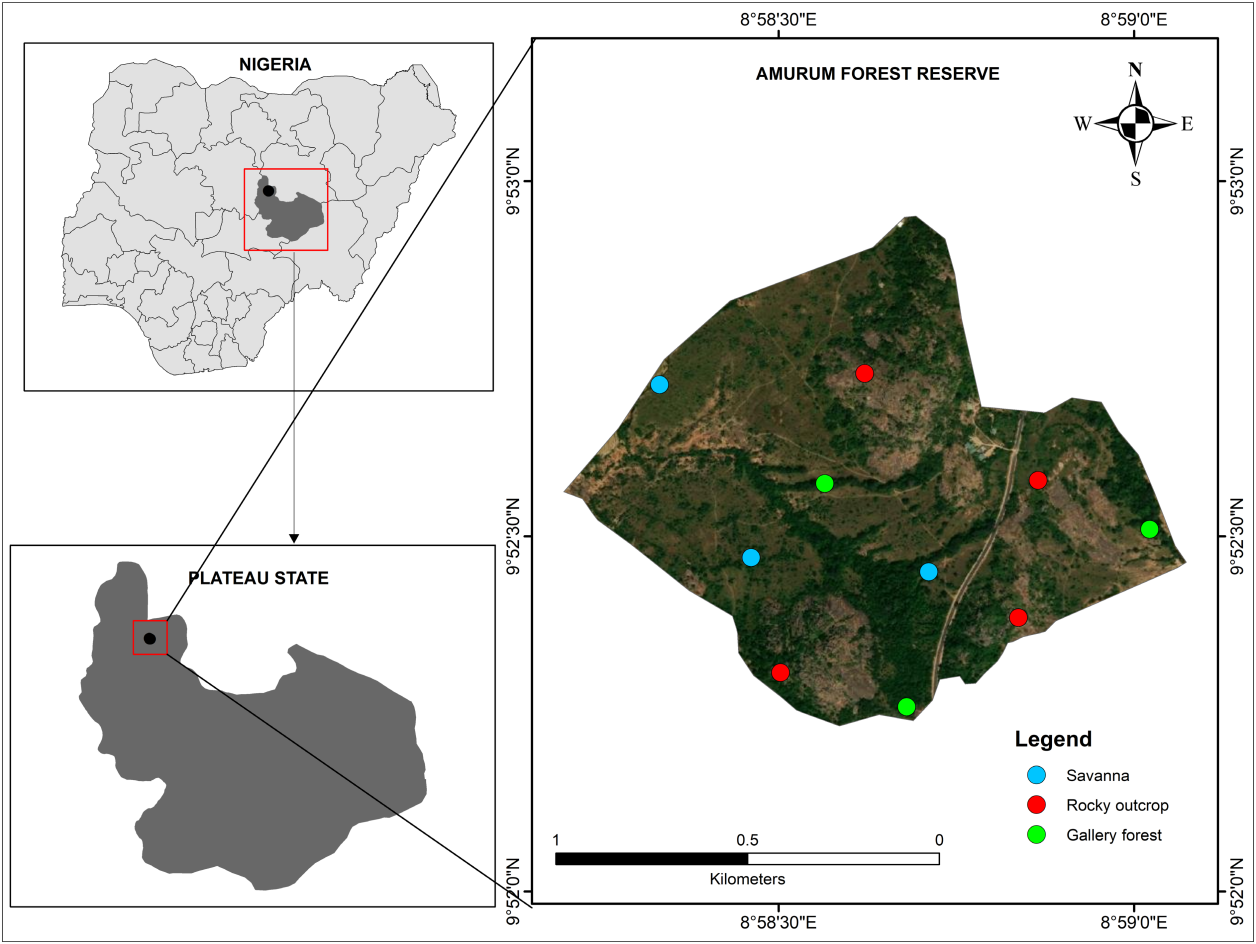
Map of the study area showing the points surveyed across the habitat types within the Amurum Forest Reserve in Plateau State of the north‐central region of Nigeria.

We conducted this study in and around the Amurum Forest Reserve (09°53′ N, 08°59′ E; Figure 1), Laminga Jos‐East, Plateau State, Nigeria. This Reserve is located within the Guinea savannah ecological zone at an altitude of about 1300 m a.s.l, with a mean annual rainfall of about 1411 mm (Daru et al., 2015; Yilangai et al., 2015). The Reserve is an Important Bird and Biodiversity Area (IBA) with over 350 species of birds including the regional endemics Jos‐plateau Indigobird *Vidua maryea* and Rock Firefinch *Lagonosticta sanguinodorsalis* (Agaldo, 2019). It also serves as a stopover and wintering site for many migrant bird species (Daru et al., 2015). Some economically important trees such as the *Pakia biglobosa*, *Lophira lanceolate*, *Cannarium* spp, *Vitex doniana* and *Khaya senegalensis* are also found within and around the reserve (Ezealor, 2002).

The Reserve has three major habitat types: gallery forest, savannah and rocky outcrops (Dawang et al., 2015; Vickery & Jones, 2002). The gallery forest found along gullies within the Reserve with seasonal water flow is characterized by dense vegetation and tall trees. The savannah habitat type is characterized mainly by grassland vegetation and interspaced with scattered trees and shrubs while the rocky outcrops are majorly made up of rocks with very sparse trees and shrubs.

The matrix surrounding the Reserve is made up of farmlands and residential areas. The predominant occupation of the people of these communities is farming, where several tons of vegetables are produced annually with the increasing number of small farms found at the edge of the Reserve (Molokwu, 2009). The crops cultivated in these communities include maize, guinea corn and cucumber. Though the Reserve is threatened by urbanization and expanding agricultural activities, it still holds remnants of natural habitats (Agaldo, 2019).

### 2.2 Survey protocol

We surveyed 20 points that were generated randomly using the Quantum Geographic Information System version 3.8 (QGIS) between November and December 2021. We ensured that these points were at least 500 m apart from each other to ensure independence following Zipkin et al. (2010). We also ensured that the points were at least 100 m away from the only major road passing through the study area to reduce the potential edge effect of the road and noise which can alter some of the techniques under comparison (Ducrettet et al., 2020).

### 2.3 Camera trap survey

We used the BrazeVideo night vision camera traps Model A252 to monitor nocturnal birds at each points from 18:00 to 06:00 h. The camera traps have a wide view angle of 110° with fully automatic IR filter that captures natural behaviours by using no glow, invisible infrared flash illumination technology equipped with 36 LEDS. The camera trap captures/detects movement within a 23 m field of view. We used a total of 40 camera traps for the survey. Two camera traps were placed at each point, one mounted at the ground level to capture understory birds and the other fixed at a height of 2.0–4.0 m to capture arboreal species (Fontúrbel et al., 2020; Moore et al., 2020). Cameras at each point were placed facing same cardinal direction.

We attached the camera traps to tree stems (natural object) because that will make them less conspicuous than when attached to poles and other non‐natural objects out in the field (Apps & McNutt, 2018; Fontúrbel et al., 2020). We also positioned the cameras in the most suitable direction to maximize view and avoid obstruction by vegetation depending on the type of habitat (Kays et al., 2020). The camera traps were operated in both photographic and video mode with a motion trigger (using three shots for a single detection and video length of 30 s).

### 2.4 Acoustic recording method

We used 20 Jolike digital omnidirectional acoustic recorders to record bird calls at each point from 18:00 to 06:00 h (astronomical dusk to dawn). The recorder has a recording bit rate of 512/1536/3071 kbps, a built‐in microphone and an independent digital signal processing (DSP) intelligent noise reduction that greatly reduces background noise and automatically detects sound. We attached the recorders to the same trees as the camera traps at an approximate height of 1.5 m above the ground level to reduce acoustic obstructions (Ducrettet et al., 2020).

### 2.5 Point count survey

During the point count survey, we visited each of the 20 points twice daily (18:00–23:59 h; 00:00–05:59 h). We spent 15 min recording only nocturnal birds seen and heard at each point during each survey. A pair of night vision binoculars (Stilnend, 3.5‐7X) alongside the Birds of Western Africa (Borrow & Demey, 2014) were used to aid bird identification. We made our observations within a 200 m radius (Lee & Marsden, 2008) with the observer facing the cardinal point which the camera traps were facing at each point. We also conducted the point count survey at least 5 m away from the location where camera traps and acoustic recorders were placed to avoid impacts on acoustic recordings quality from noises made by observer's movement (Bombaci & Pejchar, 2019). We classified species detected by point count into those that were seen and those that were heard to compare the detections of species heard during point count and acoustic recorder.

### 2.6 Analysis of recordings and pictures from camera traps

Acoustic files which were already in WAV were downloaded from the recorders and converted into spectrograms with the open‐source software Audacity version 3.1.1 (https://www.audacityteam.org/). The software was used to listen to all audio files (Fischer et al., 2023). To eliminate inter‐observer bias, the same observer (JYH) who conducted point count survey in the field analysed the acoustic recordings taking note of only nocturnal birds and the time the birds made the calls so that we can overlap them with the same 15‐min period of the point count survey. When more than one member of a species could be heard vocalizing at once, they were counted as separate individuals (Vold et al., 2017). Bird calls from Bird of Africa available in the A. P. Leventis Ornithological Research Institute (APLORI) and Xeno‐canto (https://www.xeno‐canto.org/) were used to aid the identification of bird calls (Nguyen, 2017). We only used the recordings that overlapped with the timing of the point count survey.

Pictures and videos were also extracted from the camera traps and viewed using a computer by the same observer (JYH). The pictures and videos were downloaded for the time overlapping with the point count survey. The images and videos were downloaded daily and watched and species were identified. Our camera traps captured 13,239 photographs during the study. Of these photographs, 13,095 (98.9%) were blurred images taken by the camera while 52 were *Felis catus*, 67 *Canis domestica* and 9 were *Genetta* thierryi. Also, six captures were snakes and 10 captures were rodents which we did not identify at species level.

### 2.7 Statistical analysis

We removed camera trap method from further analysis as our camera traps gave blurry photos of birds that were unidentifiable. Using descriptive statistics, we compared the species richness between the remaining survey methods, point count and acoustic recorders, and across habitat types. To determine the sampling effectiveness of the survey methods across the habitat types within the Reserve, we focused on the 10 points that were within the Reserve. Following the method by Fontúrbel et al. (2020), we calculated the expected species richness (S_est_) using Ace, Chao1, Jackknife1 and Bootstrap estimators with the SpadeR package (Chao et al., 2016). Each of these methods (Ace, Chao1, Jackknife1 and Bootstrap species richness estimators) provides species richness estimates (S_est_) based on the frequencies of unique and duplicate species, taking into account undetected species. To ensure the accuracy of the estimated species richness (S_est_), we took the average from the estimates provided by the four species richness estimators (Fontúrbel et al., 2020). We estimated the sampling effectiveness of the survey methods for each habitat type by calculating the ratio between the observed (S_obs_) and expected (S_est_) species richness (Fontúrbel et al., 2020).

$$\text{Sampling effectiveness}=\frac{\text{Observed species}\;\left(S_{\mathrm{obs}}\right)}{\text{Expected species}\;\left(S_{\mathrm{est}}\right)}*100.$$

We used the analysis of similarities (ANOSIM) to compare community composition (proportion of species present) between survey methods and habitat types. ANOSIM is a permutational non‐parametric method to compare community composition based on similarity matrices (Clarke, 1993). We estimated *p*‐values after 9999 permutations using the Bray–Curtis similarity coefficient (Fontúrbel & Jiménez, 2014). We then used the Sørensen index (QS) to estimate species similarity between the sampling methods for each habitat type. The Sørensen index (QS) is calculated as:

$$\mathrm{QS}=\frac{2*\mathrm{EC}}{A+B}$$

where EC = number of species recorded by both methods. *A* = number of species recorded by point count. *B* = number of species recorded by acoustic method. All statistical analyses were conducted using the R statistical software, Version 4.1.2 (R Core Team, 2021).

## 3 RESULTS

We recorded 11 species of nocturnal birds belonging to three families (Caprimulgidae, Strigidae and Tytonidae). Two partial migrants were recorded (Table 1): plain nightjar (*Caprimulgus inornatus*) and long‐tailed nightjar (*Caprimulgus climacurus*) while nine were resident bird species. Apart from the Verreaux's eagle owl (*Ketupa lacteaus*) that was recorded by the acoustic recorder alone, all 10 species were encountered both by the point count and the acoustic recorder methods.

**TABLE 1:** List of nocturnal avian species recorded by the three survey methods in the study area and their migratory status.

S/no.	Species	Scientific name	Family	Migration status	Point count	Acoustic recorder	Camera trap
1	African Scops Owl	*Otus senegalensis*	Strigidae	Resident	✓	✓	–
2	Black‐shouldered Nightjar	*Caprimulgus nigriscapularis*	Caprimulgidae	Resident	✓	✓	–
3	Freckled Nightjar	*Caprimulgus tristigma*	Caprimulgidae	Resident	✓	✓	–
4	Grayish Eagle‐Owl	*Buba cinerascens*	Strigidae	Resident	✓	✓	–
5	Long‐tailed Nightjar	*Caprimulgus climacurus*	Caprimulgidae	Partial migrant	✓	✓	–
6	Marsh Owl	*Asio capensis*	Strigidae	Resident	✓	✓	–
7	Northern white‐faced owl	*Ptilopsis leucotis*	Strigidae	Resident	✓	✓	–
8	Verreaux's Eagle Owl	*Ketupa lacteaus*	Strigidae	Resident	–	✓	–
9	Plain Nightjar	*Caprimulgus inornatus*	Caprimulgidae	Partial migrant	✓	✓	–
10	Standard‐winged Nightjar	*Caprimulgus longipennis*	Caprimulgidae	Resident	✓	✓	–
11	Western Barn Owl	*Tyto alba*	Tytonidae	Resident	✓	✓	–

### 3.1 Comparison of species richness between the methods

**FIGURE 2:**
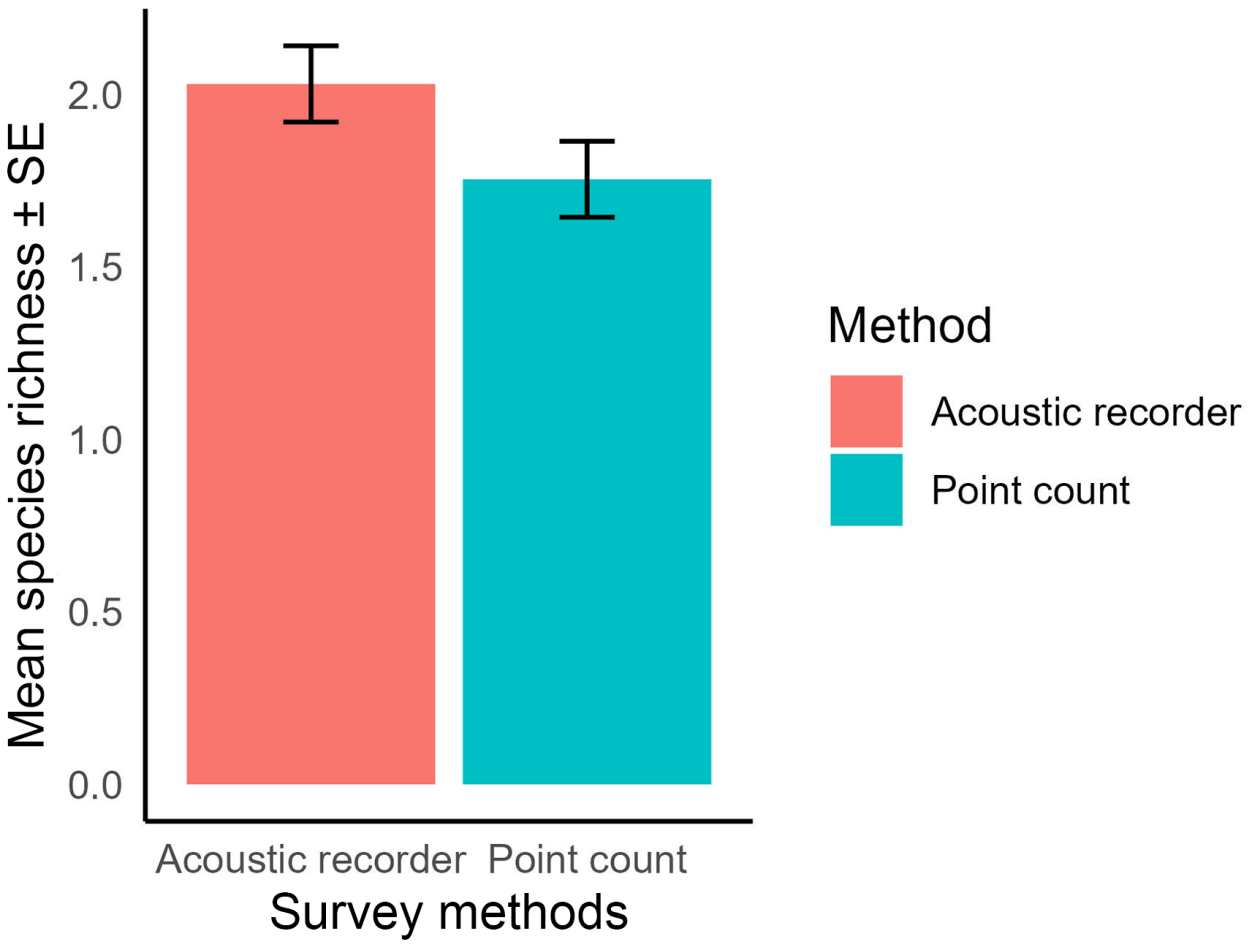
Comparison of nocturnal bird species richness between point count and acoustic recorder.

**FIGURE 3:**
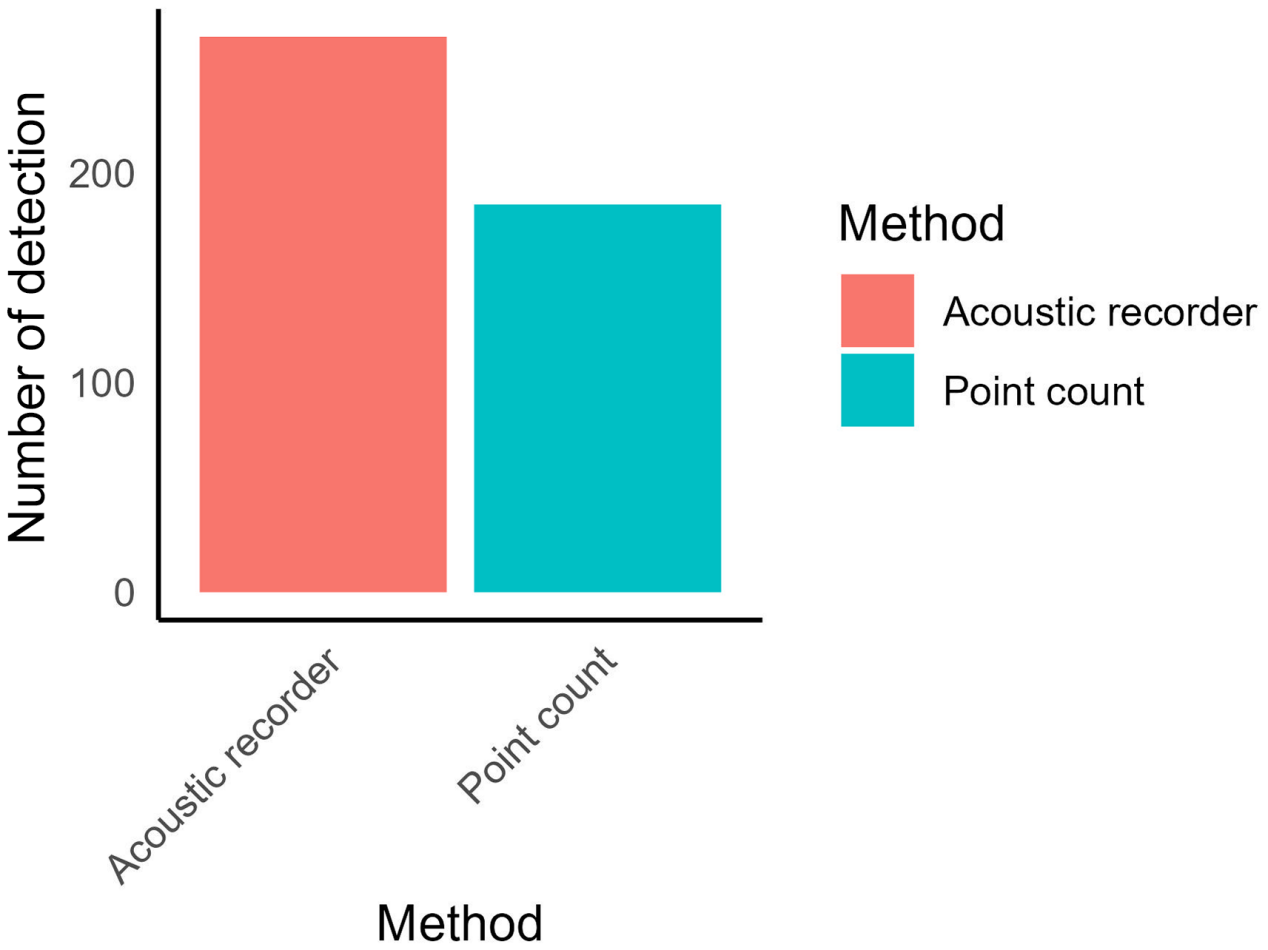
Comparison of detection frequency: Point count heard vs acoustic recorder.

The result obtained from bird species richness comparison between survey methods revealed that the two methods yielded similar mean species richness (Figure 2). However, mean bird species richness by acoustic recorder was higher (2.02 ± 0.11, mean ± SE) than the point count (1.75 ± 0.11, mean ± SE). Furthermore, acoustic recorder had a higher detection than the point count detection (Figure 3).

### 3.2 Comparison of species richness and sampling effectiveness of the survey methods across habitat types

**FIGURE 4:**
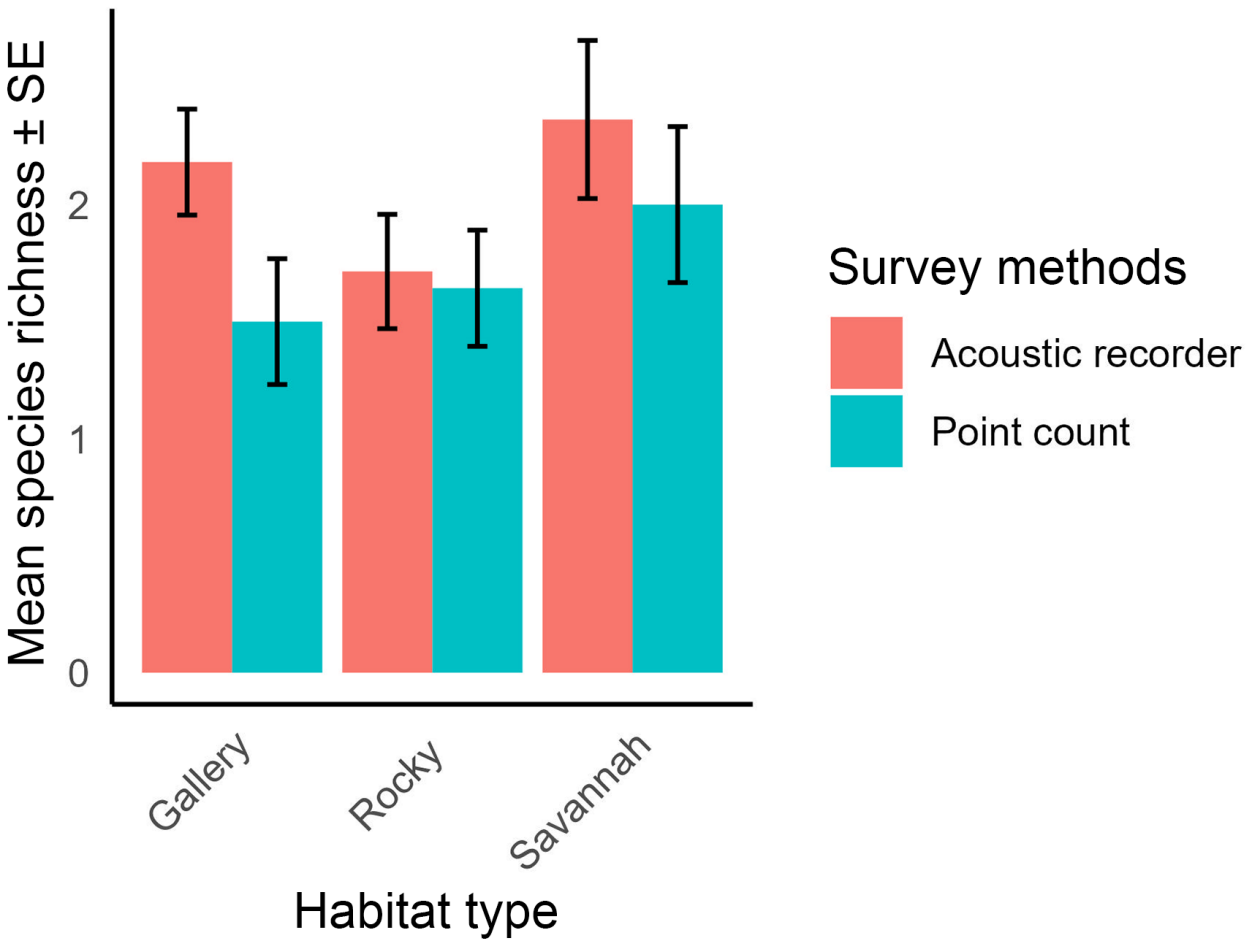
Nocturnal bird species richness across habitat types and survey methods.

We found variation in the comparison of the survey methods across habitat types in assessing nocturnal bird species richness. In the various habitat types, the mean richness obtained by acoustic recorder was higher than that of the point count (Figure 4). In the gallery forest, the acoustic recorder had higher mean richness (2.18 ± 0.22, mean ± SE) compared to point count (1.50 ± 0.26, mean ± SE). Similarly in the rocky habitat, acoustic recorder also had a higher mean species richness (1.71 ± 0.24, mean ± SE) compared to the point count (1.64 ± 0.24, mean ± SE) and the point count yielded lower mean richness (2.00 ± 0.33, mean ± SE) as compared to the acoustic recorder in the savannah (2.36 ± 0.33, mean ± SE).

For point counts and acoustic recorders, sampling effectiveness per habitat ranged from 80% to 85% and 85% to 93%, respectively (Table 2). Acoustic recorders had similar sampling effectiveness with the point count within the rocky outcrop. However, when we compared its sampling effectiveness within the gallery forests and savannah, the acoustic recorders were more effective than the point count.

**TABLE 2:** Observed (S_obs_) and expected (S_exp_) bird species richness and sampling effectiveness at three habitat types, measured using point counts and acoustic recorder.

Method	Habitat type	S_obs_	S_exp_	Effectiveness (%)
Point count	Gallery forest	7	9.33	75
	Rocky	7	8.53	82
	Savannah	8	9.99	80
Acoustic recorder	Gallery forest	7	8.87	78
	Rocky	8	8.93	89
	Savannah	9	9.35	96

Nocturnal avian composition was similar across the point count and acoustic recorder methods (ANOSIM *R* = −.01, *p* = .77) and across habitat types (ANOSIM *R* = .01, *p* = .26). Point count and acoustic recorder methods showed a similarity of 95% (Sørensen index, QS = 0.95). Examining the different habitat types, we found the highest similarity at the savannah (QS = 1), followed by the gallery forest (QS = 0.95) and finally the rocky outcrop (QS = 0.85).

## 4 DISCUSSION

Providing effective and efficient methods for sampling nocturnal birds is pivotal in understanding the distribution patterns of nocturnal species. We provide the first study that tests the sampling effectiveness of three methods in sampling nocturnal birds in the Afrotropical environment. The evidence from this study revealed that both the acoustic recorder and point count method were more effective than the camera trap technique in ascertaining the richness of nocturnal birds. Our results showed that acoustic recorder could provide similar nocturnal bird species richness as those obtained from point count. However, relatively higher sampling effectiveness with acoustic recorder suggests that the acoustic recorders can outperform the point count even across the various habitat types especially within the gallery forest and savannah.

Our result suggests that there are no perfect sampling methods for nocturnal birds especially in the comparison of acoustic recorders and point count. However, both methods provide a plethora of advantages and disadvantages that can influence the researcher's choice. Point count surveys may pose significant challenges especially in species‐rich areas where there is a high possibility of birds being overlooked especially when they call simultaneously, a scenario that was relatively common especially at dawn or the first minutes of the studies (Hutto & Stutzman, 2009). However, acoustic recorders have the advantage of being listened to over and over again, thus giving the researcher the extra time of listing all calls heard even when several species are calling simultaneously. Also, field surveys using acoustic recorders can be carried out at both larger spatial and temporal scales simultaneously (Blumstein et al., 2011; Celis‐Murillo et al., 2009; Goyette et al., 2011). Furthermore, there is the challenge regarding safety and security in conducting point count especially for nocturnal birds that are active during the night and ease of assessing study sites at such periods. This issue can be effectively addressed, particularly through the utilization of passive acoustic methods (PAM) as it offers a viable solution for tackling this problem.

Our findings demonstrate that acoustic recorder and point count have similar sampling effectiveness in assessing Afrotropical nocturnal avian communities, which is in tandem with findings in temperate regions (Frommolt & Tauchert, 2014; Zwart et al., 2014) and has already been established with the diurnal avian community (Darras et al., 2018, 2019; Wheeldon et al., 2019; Zwart et al., 2014).

Camera traps are similar to the acoustic recorder method in that they remove the effect of human /observer presence (Buxton et al., 2018). However, our camera trap did not record any nocturnal birds. The result we obtained was in contrast to previous work by Fontúrbel et al. (2020) where camera traps provided same species richness estimations with point count for avian community. The result obtained is probably due to some limitations with the camera traps as they were unidirectional compared to point count and acoustic recorders that are omnidirectional. Study‐specific biases such as differences in survey range as the camera trap (23 m field of view) as compared to point count (200 m radius) and acoustic recorder (200 m) might have a great influence. Our camera traps captured other vertebrates such as reptiles and mammals with about 200 (1.51%) of the images from the camera traps being images of birds' body parts and blurry swift flight movements which could not be identified at species level. This suggests that the limited view of the camera trap might be most effective for medium to large terrestrial animals that may be less swift in their movements (Austad, 2021; Bessone et al., 2020; Glen et al., 2013; Khwaja et al., 2019; Meek et al., 2015). Also, some other studies suggested that camera traps are mostly effective for studying larger ground‐dwelling birds but not canopy and sub‐canopy avian species (Jean‐Pierre et al., 2022; Vargas‐Daza et al., 2023); another possible explanation as to why our camera traps had no capture of owls or nightjars.

The various sampling methods available only provide a fragment of the actual avian diversity as every method with an advantage is not without limitations. Though point count is one of the widely used census technique for assessing avian community (Bibby et al., 2000; Cumming & Henry, 2019), it is not without disadvantages and limitations. Some of the limitations to point count include requirement for workforce as the method is field demanding and requires certain level of expertise observer to obtain reliable results (Ralph et al., 1995; Thomas & Marques, 2012), therefore lack of trained personnel to conduct surveys is a major setback (Holmes et al., 2014). Furthermore, the presence of an observer during point count surveys may cause disturbance and reduction in the detection rates (Digby et al., 2013).

The acoustic method is less demanding in field work and requires less expertise as recorders can be deployed by almost anyone. One of the notable advantages is that as a non‐intrusive method, it provides low level of observer interference. Despite these advantages, the method is not without some drawbacks. The use of acoustic recorders may be biased especially towards birds that are highly sonant causing species that do not call often to have a lower detection probability (Vold et al., 2017). However, recording all through the night provides a high chance of recording even quitter species when they eventually vocalize. This results in the production of huge datasets which will require complex big data processing and analytics to filter interesting sounds. Another limitation of the use of acoustic recorders is in relation to data storage. Power poses another difficulty because the system needs to be maintained on a regular basis. The unavailability of algorithm for the automatic detection of most African bird species can make analysing huge data sets from the region time‐consuming (Crunchant et al., 2020). In recent years, major improvement has been achieved for detecting species from acoustic data with the use of algorithm. However, this algorithm supports bird species from the global north and are unable to accurately detect most Afrotropical bird species presently. Other limitations include the cost of acquiring recorders and difficulty in estimating wildlife density (Pérez‐Granados & Traba, 2021).

Finally, the result we obtained from contrasting the sampling effectiveness of the methods (point count and acoustic recorder) in estimating nocturnal bird richness across different habitat types in the Amurum Forest Reserve (gallery forest, rocky and savannah) showed that the methods performed in similar way in estimating nocturnal bird richness. This suggests that the methods' performance was not affected by the type of habitat the research is being conducted which is in contrast to the prediction that habitat characteristics such as vegetation variables might influence the sampling effectiveness of the survey methods.

## 5 CONSERVATION IMPLICATIONS

There is an urgent need to prioritize conservation actions in order to monitor species loss especially for nocturnal avian species that are poorly studied in tropical Africa. The development of an efficient system for surveying animals that guarantees researcher's safety, particularly in isolated places and areas that are difficult to access during the night is crucial.

The study has revealed that acoustic recorders are as effective as other traditional methods such as point count for conducting surveys of nocturnal bird communities in a tropical African environment. The use of acoustic recorders in sampling nocturnal bird communities is a non‐intrusive method that not only guarantees researcher's safety but it also reduces the time and energy exerted during remote fieldwork (Zwart et al., 2014). The ability to use several acoustic recorders simultaneously over a long period of time across a large spatial area with reduced supervision will allow for it to be employed in bird monitoring such as PAM. The rapid advancements in biomonitoring methods over the past 10 years have changed how we remotely study the distribution of wildlife, allowing us to better plan surveys, identify hotspots and monitor how wildlife reacts to ever‐increasing anthropogenic disturbance to their environments. O'Bryan et al. (2022) revealed that nocturnal species such as owls are lacking critical information on the factors driving them to extinction. While the use of acoustic recorded especially in the Afrotropics could provide insight into other aspect of their ecology such as habitat preference, distribution and behaviour, it is worth noting that the choice of survey method should be dependent on the research questions, preference and species‐specific study. These insights will be pivotal in the conservation of nocturnal species.

## AUTHOR CONTRIBUTIONS

**Yitmwa Hope Joel:** Data curation (lead); formal analysis (lead); methodology (equal); project administration (supporting); writing – original draft (lead); writing – review and editing (supporting). **Iniunam Aniefiok Iniunam:** Data curation (supporting); formal analysis (supporting); methodology (equal); project administration (supporting); writing – original draft (supporting); writing – review and editing (supporting). **Danjuma Filibus Dami:** Methodology (equal); supervision (supporting); writing – review and editing (supporting). **Ulf Ottosson:** Methodology (equal); supervision (supporting); writing – review and editing (supporting). **Adams Adamanyiwa Chaskda:** Conceptualization (lead); funding acquisition (lead); methodology (equal); project administration (lead); supervision (lead); writing – review and editing (lead).

## CONFLICT OF INTEREST STATEMENT

The authors have no conflict of interest.

## Supporting information


Data S1.

## Data Availability

All data are available at the Nocturnal Avian Ecology Lab at the A.P Leventis Ornithological Research Institute (APLORI) and will be made available upon request.
